# Adversarially Learned Total Variability Embedding for Speaker Recognition with Random Digit Strings

**DOI:** 10.3390/s19214709

**Published:** 2019-10-30

**Authors:** Woo Hyun Kang, Nam Soo Kim

**Affiliations:** Department of Electrical and Computer Engineering and the Institute of New Media and Communications, Seoul National University, Seoul 08826, Korea; whkang@hi.snu.ac.kr

**Keywords:** speech embedding, deep learning, speaker recognition, unsupervised representation learning

## Abstract

Over the recent years, various research has been conducted to investigate methods for verifying users with a short randomized pass-phrase due to the increasing demand for voice-based authentication systems. In this paper, we propose a novel technique for extracting an i-vector-like feature based on an adversarially learned inference (ALI) model which summarizes the variability within the Gaussian mixture model (GMM) distribution through a nonlinear process. Analogous to the previously proposed variational autoencoder (VAE)-based feature extractor, the proposed ALI-based model is trained to generate the GMM supervector according to the maximum likelihood criterion given the Baum–Welch statistics of the input utterance. However, to prevent the potential loss of information caused by the Kullback–Leibler divergence (KL divergence) regularization adopted in the VAE-based model training, the newly proposed ALI-based feature extractor exploits a joint discriminator to ensure that the generated latent variable and the GMM supervector are more realistic. The proposed framework is compared with the conventional i-vector and VAE-based methods using the TIDIGITS dataset. Experimental results show that the proposed method can represent the uncertainty caused by the short duration better than the VAE-based method. Furthermore, the proposed approach has shown great performance when applied in association with the standard i-vector framework.

## 1. Introduction

Over the last decade, the i-vector framework [1,2] has become one of the most dominant approaches for feature extraction in text-independent speaker recognition. The widespread popularity of the i-vector framework in the speaker recognition community can be attributed to its ability to map the distributive pattern of speech with various duration to a fixed dimensional vector. The i-vector framework, along with many other classical utterance-level speech representation methods (e.g., eigenvoice adaptation and joint factor analysis) [3,4], focuses on efficiently compressing the Gaussian mixture model (GMM) supervector, which is formed by concatenating the mean vector of each mixture component [5], via linear factorization. However, due to its linear nature, the i-vector framework may not describe the whole variability of the given speech utterance.

To represent the variability which could not be expressed through linear processing, various attempts have been made utilizing deep neural networks (DNNs) in learning the nonlinear relationship between the input and the output for feature extraction. Most recent works in deep learning-based feature extraction have focused on nonlinearly modeling the frame-level characteristics in a similar manner to the DNN-based acoustic modeling in automatic speech recognition (ASR) [6].

In [7], a DNN for frame-level speaker classification is trained and the activations of the last hidden layer are taken as a nonlinear speaker representation. In [8,9], a TDNN (time-delay neural network)-based utterance embedding technique is proposed, where the embedding is obtained by aggregating the frame-level activations of the TDNN. In [10,11], the speaker embedding neural networks are optimized to directly minimize the verification loss in an end-to-end fashion. Although the conventional deep learning-based feature extraction schemes have shown the potential of using nonlinearly extracted features in speaker recognition, they are usually trained in a supervised manner and it is almost impossible to apply them when no labeled data are available for training.

Recently, verifying users with randomized pass-phrase with constrained vocabulary has become an important task due to the increasing demand for voice-based authentication systems [12]. In this technique, the users are enrolled and verified by speaking random digit strings. One of the obstacles in random digit string task is the feature uncertainty caused by the short duration of the spoken utterance [13]. Since a critical amount of speaker relevant information is contained in the phonetic characteristics, the lack of phonetically informative frames can lead to degradation of the quality of the extracted utterance-level feature. It has been reported that the conventional i-vector framework suffers from severe performance deterioration when short duration speech is applied in the verification process [14,15,16]. In real-life applications, this problem should be considered more carefully as the speech samples for enrollment and trial are required to be short.

In our previous work [17], we successfully exploited the variational autoencoder (VAE) framework [18,19] to extract an utterance-level feature to capture the total variability and the uncertainty of the speech in a nonlinear fashion. The VAE has an autoencoder-like structure and assumes that the input data are generated through a directed graphical model induced by a latent variable. The latent variable of the VAE-based feature extraction model in [17] serves as an utterance-level representation and has shown better performance than the conventional i-vector in coping with the uncertainty caused by the short duration. Moreover, we could observe that the variance of the latent variable can be used as a proxy for the uncertainty caused by the short duration of the given speech samples. However, as mentioned in [20,21], since the Kullback–Leibler divergence (KL divergence) regularization term in the VAE objective function focuses on stretching the latent variable space over the entire training set to avoid assigning a small probability to any samples, the VAE output often lacks variety. This may lead the VAE-based feature extractor to yield utterance-level features which lack distinct speaker-dependent information, resulting in limited performance of the overall speaker recognition system.

In order to overcome this problem, we propose a novel approach to train a deep learning-based feature extraction network using the adversarial learning framework. The proposed method adopts an adversarially learned inference (ALI) model [22] to nonlinearly express the total variability and uncertainty of the given speech. Analogous to the VAE-based feature extractor in [17], the proposed model is trained according to the maximum likelihood criterion and the latent variable serves as the utterance-level feature representation. However, instead of simply regularizing the latent variable via KL divergence, the proposed method uses a discriminator network to make sure that the generated latent variable and GMM supervector are close to the latent prior distribution and the GMM obtained through maximum a posteriori (MAP) adaptation, respectively. While training the proposed network, the parameters of the feature extraction and the discriminator networks are updated competitively; the feature extraction network tries to generate a more realistic GMM supervector and latent variable while the discriminator network focuses on distinguishing the generated GMM and latent variable from the real ones. By training the feature extraction network in this adversarial fashion, the proposed system is expected to provide utterance-level features that can capture more prominent speaker relevant characteristics and the uncertainty within the given speech utterance. Similarly to the VAE-based feature extractor, the utterance-level features extracted from the proposed model can substitute the conventional i-vector extraction module without any difficulty.

To evaluate the performance of the proposed method, along with the VAE-based feature extraction scheme and the conventional i-vector framework in the random digits task, we conducted a set of speaker verification experiments using the TIDIGITS dataset. Experimental results show that the proposed method outperforms the standard i-vector framework and the VAE-based method in terms of equal error rate (EER). It is also interesting to see that impressive performance improvement is observed when the features extracted from the proposed method and the conventional i-vector are augmented together. This implies that the feature extracted using the proposed method is complementary to that extracted from the conventional i-vector framework.

The rest of this paper is organized as follows: We first briefly describe the basic ideas and important formulations of the conventional i-vector and the ALI frameworks in Section 2. In Section 3, the newly proposed ALI-based feature extractor and its training scheme are presented. The experiments and results are shown in Section 4. Finally, Section 5 concludes the paper.

## 2. Related Work

### 2.1. I-Vector Framework

Once a universal background model (UBM) is provided, which is a GMM representing the utterance-independent distribution of the frame-level features, an utterance-dependent GMM can be attained by adjusting the UBM parameters using the maximum a posteriori (MAP) adaptation algorithm [23]. By concatenating the mean vector of each Gaussian mixture component, a GMM supervector can be obtained, which summarizes the overall pattern of the frame-level feature distribution. However, using the GMM supervector as an utterance-level feature may limit the overall speaker recognition performance due to its high dimensionality.

To solve this problem, various methods for reducing the dimensionality of the GMM supervector have been proposed [1,3,4]. Specifically, the i-vector framework is now widely used to represent the idiosyncratic characteristics of the utterance in speaker and language recognition [24]. The extraction of an i-vector can be viewed as a factorization process decomposing the GMM supervector as

(1) m(X)=u+Tw(X)

where m(X), u, T, and w(X) indicate the ideal GMM supervector dependent on a given speech utterance X, UBM supervector, total variability matrix, and i-vector, respectively. The i-vector framework aims to find the optimal w(X) and T to adapt the UBM parameters to a given speech utterance X. Given X, the zeroth- and the first-order Baum–Welch statistics are obtained as

(2) $$n_{c}\left(X\right)=\sum_{l=1}^{L}\gamma_{l}\left(c\right)$$

(3) $${\tilde{f}}_{c}\left(X\right)=\sum_{l=1}^{L}\gamma_{l}\left(c\right)\left(x_{l}-u_{c}\right)$$

where, for each frame *l* within X with *L* frames, $\gamma_{l}\left(c\right)$ is the posterior probability that the *l*th frame feature $x_{l}$ is aligned to the *c*th mixture component of the UBM, $u_{c}$ is the mean vector of the *c*th mixture component of the UBM, and $n_{c}\left(X\right)$ and ${\tilde{f}}_{c}\left(X\right)$ are the zeroth- and the centralized first-order Baum–Welch statistics, respectively.

**Figure 1:**
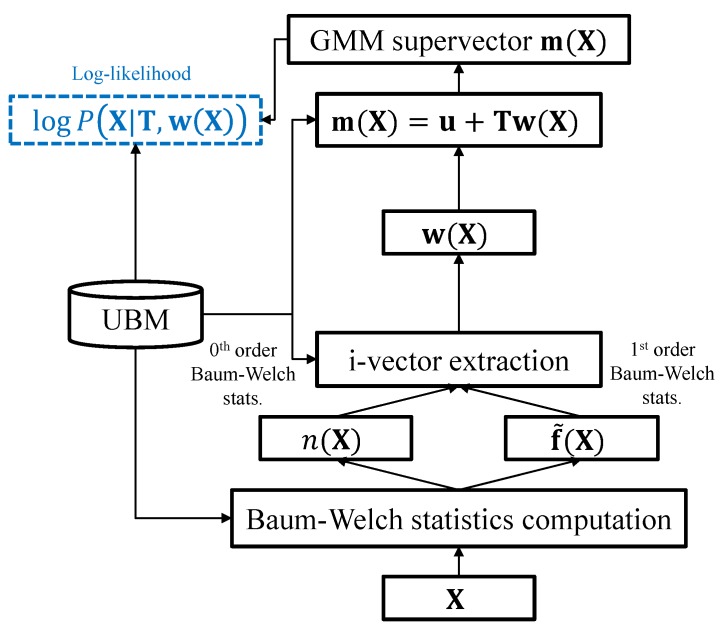
Flow chart of the i-vector framework. Note that when training the i-vector extractor, the GMM supervector is not explicitly computed. The Baum–Welch statistics computation stage is described in Equations (2) and (3), and the log-likelihood formulation is described in Equation (5).

As shown in Figure 1, the i-vector framework can be considered as an adaptation process where the mean of each UBM mixture component is adjusted to maximize the likelihood with respect to a given utterance, and the estimated i-vector is served as the adaptation factor. Let $\Sigma_{c}$ denote the covariance matrix of the *c*th UBM mixture component and *F* be the dimensionality of the frame-level features. The posterior probability $\gamma_{l}\left(c\right)$ is computed as follows:

$$\begin{array}{c} \gamma_{l}\left(c\right)=\frac{\pi_{c}N\left(x_{l}|u_{c},\Sigma_{c}\right)}{\sum_{i=1}^{C}\pi_{i}N\left(x_{l}|u_{i},\Sigma_{i}\right)} \end{array}$$

where $\pi_{c}$ is the weight of the *c*th Gaussian mixture of the UBM. The log-likelihood given an utterance X conditioned on w(X) can be obtained as

(4) $$\log P\left(X|T,w\left(X\right)\right)=\sum_{c=1}^{C}\left(n_{c}\left(X\right)\log\frac{1}{{\left(2\pi \right)}^{F/2}{\left|\Sigma_{c}\right|}^{1/2}}-\frac{1}{2}\sum_{l=1}^{L}\gamma_{l}\left(c\right){\left(x_{l}-m_{c}\left(X\right)\right)}^{t}\Sigma_{c}^{-1}\left(x_{l}-m_{c}\left(X\right)\right)\right)$$

where $m_{c}\left(X\right)$ is the mean of the *c*th mixture component of m(X) and the superscript *t* indicates matrix transpose. The log-likelihood given X is obtained by marginalizing (4) over w(X) as

(5) $$\log P\left(X|T\right)=\log E_{w}\left[P\left(X|T,w\right)\right]=\log\int P\left(X|T,w\right)N\left(w|0,I\right)dw$$

The total variability matrix T is trained to maximize the log-likelihood (5) via the expectation– maximization (EM) algorithm. Interested readers are encouraged to refer to [1,2] for further details of the i-vector framework.

### 2.2. Adversarially Learned Inference

**Figure 2:**
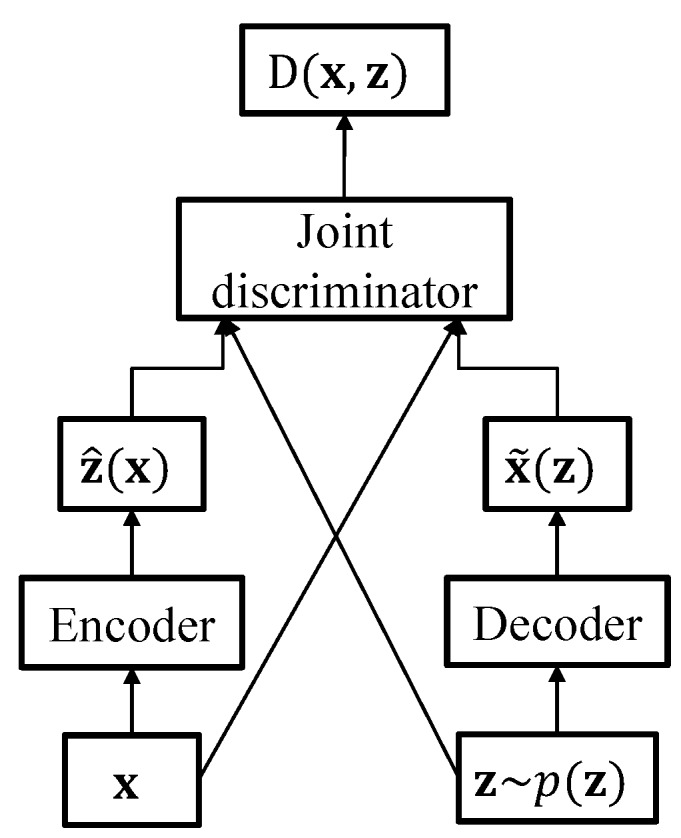
Flow chart of the ALI framework. The encoder, decoder and joint discriminators are feed-forward neural networks, each consisting of nonlinear hidden layers.

Like other variants of the generative adversarial network (GAN) [25], ALI aims to train a network for generating a realistic data sample with the help of a discriminator network, which tries to predict whether the input data are real or generated [22]. However, unlike the ordinary GAN framework that cannot analyze the data at an abstract level, ALI integrates an inference network for estimating the random latent variable z from the input data. As depicted in Figure 2, ALI is composed of three directed networks: encoder, decoder, and joint discriminator networks. Analogous to the VAE network, the encoder network outputs the mean and variance of the posterior distribution p(z|x) given an observation x. On the other hand, the decoder generates the data sample from a latent variable sampled from a prior distribution p(z).

The encoder and decoder networks represent the joint probability distributions of the latent variable z and the observed data x as follows:

(6) $$q_{\phi }\left(x,z\right)=q_{\phi }\left(x\right)q_{\phi }\left(z|x\right),p_{\theta }\left(x,z\right)=p_{\theta }\left(z\right)p_{\theta }\left(x|z\right)$$

In (6), ϕ, θ, $q_{\phi }\left(x,z\right)$, and $p_{\theta }\left(x,z\right)$ denote the encoder parameters, decoder parameters, and the joint distributions represented by the encoder and decoder, respectively. The encoder marginal probability $q_{\phi }\left(x\right)$ represents the real data distribution and the decoder marginal probability $p_{\theta }\left(z\right)$ is the prior distribution of the latent variable, usually specified as a standard normal distribution. The conditional probability distributions $q_{\phi }\left(z|x\right)$ and $p_{\theta }\left(x|z\right)$ represent the inferred latent distribution output by the encoder and the distribution of the data generated by the decoder, respectively.

The joint discriminator takes a joint pair of the data and the latent variable as input and distinguishes between the samples from the encoder $\left(x,\hat{z}\left(x\right)\right)∼q_{\phi }\left(x,z\right)$ and the ones from the decoder $\left(\tilde{x}\left(z\right),z\right)∼p_{\theta }\left(x,z\right)$. The discriminator output D(x,z) is sigmoidal, ideally having a value close to 0 if the samples are drawn from $p_{\theta }\left(x,z\right)$ and 1 if drawn from $q_{\phi }\left(x,z\right)$.

The encoder, decoder, and the joint discriminator networks of the ALI are trained adversarially by minimizing the following objective functions:

(7) $$E_{D}=-E_{\left(x,z\right)∼q_{\phi }\left(x,z\right)}\left[\log\left(D\left(x,z\right)\right)\right]-E_{\left(x,z\right)∼p_{\theta }\left(x,z\right)}\left[\log\left(1D\left(x,z\right)\right)\right]$$

(8) $$E_{G}=-E_{\left(x,z\right)∼q_{\phi }\left(x,z\right)}\left[\log\left(1D\left(x,z\right)\right)\right]-E_{\left(x,z\right)∼p_{\theta }\left(x,z\right)}\left[\log\left(D\left(x,z\right)\right)\right]$$

In Equations (7) and (8), $E_{D}$ denotes the discriminator loss function and $E_{G}$ is the generator loss function. The joint discriminator network is trained to minimize $E_{D}$, which decreases as the network distinguishes between the samples from the encoder and the decoder better and the parameters θ,ϕ of the generator (i.e., encoder and decoder networks) are updated to minimize $E_{G}$.

## 3. Adversarially Learned Feature Extraction

**Figure 3:**
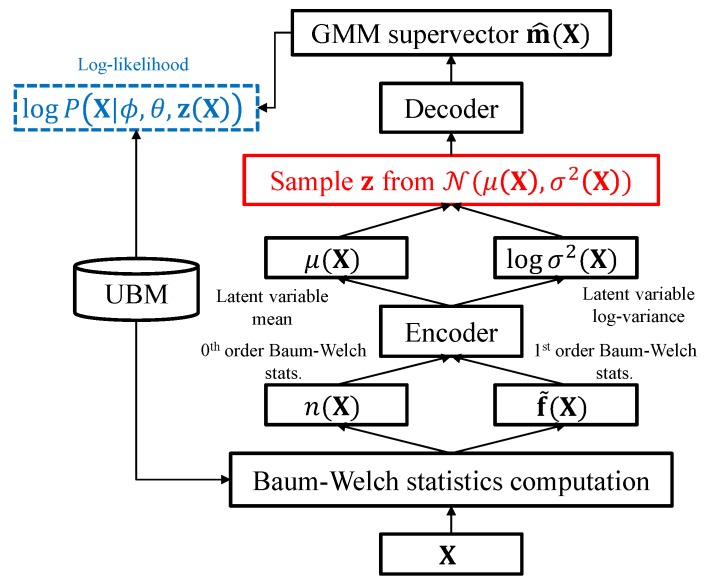
Maximum likelihood training scheme for the proposed nonlinear feature extractor. Red shows the sampling operation. Blue shows the loss term. The log-likelihood computation and sampling operation are described in Equations (10) and (13), respectively.

The proposed algorithm assumes that the variability of the utterance-dependent GMM supervector of the UBM can be represented by a nonlinear projection of a hidden variable onto the variability space as follows:

(9) m(X)=u+g(z(X))

where g is a nonlinear function which transforms the hidden variable z(X) to the total variability of the GMM supervector m(X). As shown in Figure 3, the proposed scheme employs an encoder network for inferring the hidden variable z(X) from the observed speech X and a decoder network for nonlinearly mapping z(X) onto the total variability space to generate an ideal utterance-dependent GMM supervector.

As with the i-vector adaptation framework, the main task of the proposed approach is to generate an utterance-dependent GMM that maximizes the likelihood given the Baum-Welch statistics of the utterance.The encoder network takes the zeroth- and first-order Baum-Welch statistics of the input utterance X and estimates the posterior distribution, namely the mean μ and the variance $\sigma^{2}$ of the latent variable through variational inference. The latent variable z is a random variable assumed to follow a Gaussian distribution and its components are uncorrelated with each other. The decoder network generates the GMM supervector given the latent variable according to the maximum likelihood criterion.

### 3.1. Maximum Likelihood Criterion

Analogous to (4), the log-likelihood of the GMM supervector $\hat{m}\left(X\right)$ generated from the decoder network conditioned on the latent variable z(X), which is sampled from the posterior distribution $q_{\phi }\left(z|X\right)$ generated by the encoder network given input observation X, can be written as

(10) $$\begin{array}{cc} \log\; & P\left(X|\phi ,\theta ,z\left(X\right)\right)=\sum_{c=1}^{C}(n_{c}\left(X\right)\log\frac{1}{{\left(2\pi \right)}^{F/2}{\left|\Sigma_{c}\right|}^{1/2}}\\ \; & -\frac{1}{2}\sum_{l=1}^{L}\gamma_{l}\left(c\right){\left(x_{l}-{\hat{m}}_{c}\left(X\right)\right)}^{t}\Sigma_{c}^{-1}\left(x_{l}-{\hat{m}}_{c}\left(X\right)\right)) \end{array}$$

where ${\hat{m}}_{c}\left(X\right)$ denotes the *c*th component of $\hat{m}\left(X\right)$. Instead of directly maximizing the marginal log-likelihood $\log P\left(X|\phi ,\theta \right)=\log E_{z\left(X\right)∼q_{\phi }\left(z|X\right)}\left[P\left(X|\phi ,\theta ,z\left(X\right)\right)\right]$, which is practically intractable, the proposed algorithm maximizes its lower bound which can be obtained via Jensen’s inequality as follows:

(11) $$\log P\left(X|\phi ,\theta \right)=\log E_{z\left(X\right)∼q_{\phi }\left(z|X\right)}\left[P\left(X|\phi ,\theta ,z\left(X\right)\right)\right]\geq E_{z\left(X\right)∼q_{\phi }\left(z|X\right)}\left[\log P\left(X|\phi ,\theta ,z\left(X\right)\right)\right]$$

Using the reparameterization trick [18], the Monte Carlo estimate of the marginal log-likelihood lower bound can be computed as

(12) $$E_{z\left(X\right)∼q_{\phi }\left(z|X\right)}\left[\log P\left(X|\phi ,\theta ,z\left(X\right)\right)\right]≃\frac{1}{S}\sum_{s=1}^{S}\log P\left(X|\phi ,\theta ,z_{s}\left(X\right)\right)$$

where *S* is the number of samples used for the estimation, and $z_{s}\left(X\right)$ is the reparameterized latent variable defined by

(13) $$z_{s}\left(X\right)=\mu \left(X\right)+\sigma \left(X\right)\epsilon_{s}$$

In (13), μ(X) and σ(X) are respectively the mean and standard deviation of the latent variable z(X) generated from the encoder network, and $\epsilon_{s}∼N\left(0,I\right)$ is an auxiliary noise variable.

### 3.2. Adversarially Learned Inference for Nonlinear I-Vector Extraction

**Figure 4:**
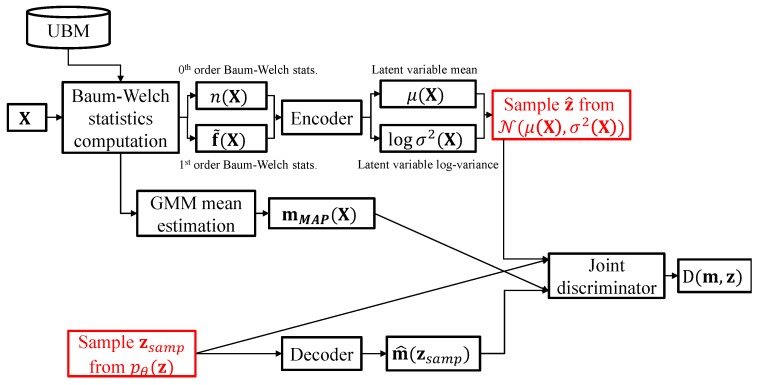
ALI-based training scheme for the proposed nonlinear feature extractor. Red shows the sampling operations. The GMM mean estimation is described in (14) and more detailed information on the joint discriminator structure is depicted in Figure 5.

In order to ensure that the generated latent variable z matches its prior distribution and the GMM supervector $\hat{m}$ well preserves the distributive structure of the GMM driven from the UBM, the proposed scheme utilizes a joint discriminator for regularizing the encoder and decoder parameters. As shown in Figure 4, the joint discriminator of the proposed algorithm takes the GMM supervector and the latent variable and tries to determine whether the input pairs are generated from the encoder or the decoder networks.

However, since the decoder output $\hat{m}\left(z\right)$ does not match the encoder inputs n(X) and $\tilde{f}\left(X\right)$, the joint discriminator cannot be applied directly. To alleviate this difficulty, the proposed scheme first estimates the GMM mean vectors via maximum a posteriori (MAP) adaptation [23] given as follows:

(14) $$m_{c,MAP}\left(X\right)=\frac{\sum_{l=1}^{L}\gamma_{l}\left(c\right)x_{l}}{\sum_{l=1}^{L}\gamma_{l}\left(c\right)}=\frac{{\tilde{f}}_{c}\left(X\right)}{n_{c}\left(X\right)}+u_{c}$$

where $m_{c,MAP}\left(X\right)$ is the estimated *c*th Gaussian mixture mean given the input speech utterance X. The mean vectors $m_{c,MAP}\left(X\right)$ for c=1,...,C are concatenated to form a GMM supervector $m_{MAP}\left(X\right)$.

The joint discriminator takes the joint pair either from the encoder $\left(m_{MAP}\left(X\right),\hat{z}\right)∼q_{\phi }\left(m,z\right)$ or from the decoder $\left(\hat{m}\left(z_{samp}\right),z_{samp}\right)∼p_{\theta }\left(m,z\right)$ as input, where $\hat{z}$ and $z_{samp}$ are the latent variables sampled from $N(\mu \left(X\right),\log\sigma^{2}\left(X\right))$ and $p_{\theta }\left(z\right)$, respectively, and $\hat{m}\left(z_{samp}\right)$ is the GMM supervector generated by the decoder given $z_{samp}$. We assume that the prior distribution for the latent variable $p_{\theta }\left(z\right)$ to be N(z|0,I), akin to the prior for w in the i-vector framework. As in (7), the discriminator network parameter is trained to minimize the following objective function:

(15) $$E_{Prop,D}=-E_{\left(m,z\right)∼q_{\phi }\left(m,z\right)}\left[\log\left(D\left(m,z\right)\right)\right]-E_{\left(m,z\right)∼p_{\theta }\left(m,z\right)}\left[\log\left(1D\left(m,z\right)\right)\right]$$

By combining the generator loss function of ALI (8) and the marginal log-likelihood lower bound (12), the objective function of the encoder and decoder networks of the proposed framework can be written as

(16) $$\begin{array}{cc} E_{Prop,G}= & -E_{\left(m,z\right)∼q_{\phi }\left(m,z\right)}\left[\log\left(1D\left(m,z\right)\right)\right]\\ \; & -E_{\left(m,z\right)∼p_{\theta }\left(m,z\right)}\left[\log\left(D\left(m,z\right)\right)\right]\\ \; & -\frac{1}{S}\sum_{s=1}^{S}\log P\left(X|\phi ,\theta ,z_{s}\left(X\right)\right) \end{array}$$

From (16), it is seen that the encoder and decoder networks are trained not only to generate latent variables and GMM supervectors from $q_{\phi }\left(m,z\right)$ or $p_{\theta }\left(m,z\right)$ that are identical to each other, but also to maximize the log-likelihood of the generated utterance-dependent GMM by minimizing $E_{Prop,G}$ through error back-propagation [26].

The proposed adversarially learned feature extraction framework is trained by alternating between the optimization of the generator (i.e., encoder and decoder network) and the discriminator every epoch. Once the networks are trained, the encoder network is used as an feature extractor, where the latent variable mean and log-variance vectors are used as nonlinear utterance-level features.

### 3.3. Relationship to the VAE-Based Feature Extractor

The VAE-based feature extraction network [17] focuses on maximizing the log-likelihood of the generated GMM by minimizing the following objective function:

(17) $$E_{VAE/FE}\left(X\right)=D_{\mathrm{KL}}\left(q_{\phi }\left(z|X\right)\|p_{\theta }\left(z\right)\right)-\frac{1}{S}\sum_{s=1}^{S}\log P\left(X|\phi ,\theta ,z_{s}\left(X\right)\right)$$

The first term in the RHS of (17) is the KL divergence between the prior and the posterior distribution of the latent variable z, which can be viewed as the regularization term. The regularization term forces the encoder network to generate a latent variable distribution that is compatible with $p_{\theta }\left(z\right)$. However, the KL regularization term stretches the latent space over the entire training set to avoid assigning small probability to any training samples [20,21]. Due to such problem of the KL regularization term, the VAE tends to generate conservative outputs, which usually lack in variety. Especially in the image processing community, it has been reported that the VAE-based image generators result in blurry image samples [22]. In the same manner, the KL regularization term may lead the VAE-based feature extractor to produce utterance-level features with insufficient idiosyncratic representation for the speaker.

The proposed ALI-based feature extraction framework, in contrast, does not regularize the latent variable distribution with a KL divergence term. Instead, the proposed scheme employs a joint discriminator network, which encourages the encoder and decoder networks to generate realistic latent variables and GMM supervectors. Thus, the distinctive information within the latent variables generated by the ALI-based feature extractor is less likely to be tightly constrained by its prior distribution.

## 4. Experiments

### 4.1. Databases

In order to evaluate the performances of the baseline systems and the proposed scheme in a condition similar to real-life application where the speech data for training and enrolling are limited and usually have short durations, we performed experiments using the TIMIT dataset [27] as the development set and TIDIGITS dataset [28] as the enrollment and trial sets. The TIMIT dataset contains 6300 clean recorded utterances, 10 utterances spoken by each of 438 male and 192 female speakers. Each utterance in the TIMIT dataset has an average duration of three seconds. The TIMIT dataset was used for training the UBM and also used for training the total variability matrix. The TIDIGITS dataset contains 25,096 clean recorded utterances spoken by 111 male, 114 female, 50 boy, and 51 girl speakers. Each of the 326 speakers in the TIDIGITS dataset spoke a set of isolated digits and 2–7 digit sequences. The TIDIGITS dataset was split into two subsets, each containing 12,548 utterances from all 326 speakers, and they were separately used as the enrollment and trial data.

### 4.2. Experimental Setup

The acoustic features used in the experiments were 19-dimensional Mel-frequency cepstral coefficients (MFCCs) and the log-energy extracted at every 10 ms, using a 20 ms Hamming window via the SPro library [29]. Together with the delta and delta–delta of the 19-dimensional MFCCs and the log-energy, the frame-level acoustic feature used in our experiments was a 60-dimensional vector.

**Figure 5:**
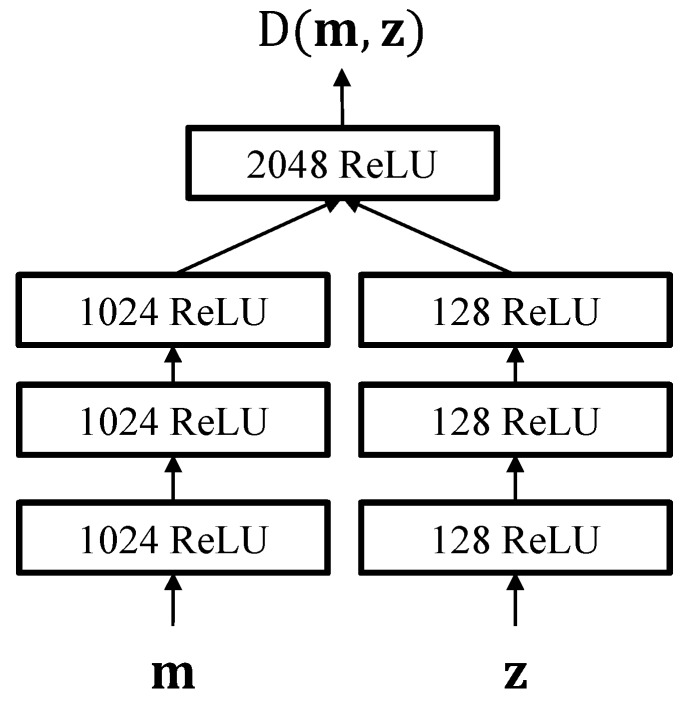
Network structure for the joint discriminator of the ALI-based feature extractor. Each block represents a hidden layer with rectified linear unit (ReLU) activation function.

We trained a UBM containing 32 mixture components in a gender-independent manner, using all the speech utterances in the TIMIT dataset. Training the UBM, total variability matrix, and the i-vector extraction were done by using the MSR Identity Toolbox via MATLAB [30]. The encoder and decoder of the experimented VAE- and ALI-based networks were configured to have a single hidden layer with 4096 ReLU nodes, and the dimensionality of the latent variables was set to be 200. As depicted in Figure 5, the discriminator network of the ALI-based feature extraction model was configured to follow a similar structure to the multimodal network [31]. The first few hidden layers model the higher-level representation of the GMM supervector and latent variable, and the last hidden layer models the joint information between them. The implementation of the experimented networks was done using Tensorflow [32] and trained using the AdaGrad optimization technique [33]. In addition, dropout [34] with a fraction of 0.8 and L2 regularization with a weight of 0.01 were applied for training all the VAEs, and the Baum–Welch statistics extracted from the entire TIMIT dataset were used as training data. A total of 100 samples were used for reparameterization to approximate the expectations in (15).

For all the extracted utterance-level features, linear discriminant analysis (LDA) [35] was applied for feature compensation and the dimensionality was finally reduced to 200. Probabilistic linear discriminant analysis (PLDA) [36] was used for speaker verification, and the speaker subspace dimension was set to be 200.

Two performance measures were evaluated in our experiments: classification error (Class. err.) and EER. The classification error was measured while performing a speaker identification task where each trial utterance was compared with all the enrolled speakers via PLDA, and the enrolled speaker with the highest score was chosen as the identified speaker. Then, the ratio of the number of falsely classified samples to the total number of trial samples represents the classification error. The EER is a widely used measure for speaker verification which indicates the error when the false alarm rate (FAR) and the false reject rate (FRR) are the same [35].

### 4.3. Effect of the Duration on the Latent Variable

In order to evaluate the effectiveness of using the latent variable variance as a measure for uncertainty caused by short duration, the differential entropy, which measures the average uncertainty of a random variable, was computed. Since the latent variable z(X) is assumed to follow a Gaussian distribution, the differential entropy can be formulated as follows:

(18) $$h\left(z\left(X\right)\right)=\frac{1}{2}\log{\left(2\pi e\right)}^{K}+\frac{1}{2}\log\prod_{k=1}^{K}\sigma_{k}^{2}\left(X\right)$$

In (18), *K* represents the dimensionality of the latent variable, and $\sigma_{k}^{2}\left(X\right)$ is the *k*th element of $\sigma^{2}\left(X\right)$.

**Figure 6:**
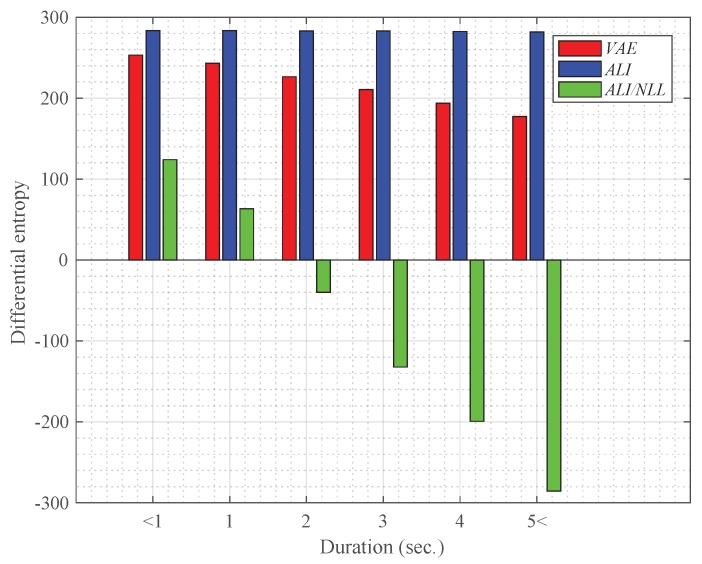
Average differential entropy computed using the latent variable variance extracted from the VAE- and ALI-based systems on different durations.

From each speech sample in the entire TIDIGITS dataset, the variance of the 200-dimensional latent variable was obtained using the encoder network and used for computing the differential entropy. As shown in Figure 6, we experimented with the latent variables extracted from three different feature extraction models:

- *VAE*: the VAE-based feature extraction network trained to minimize (17)
- *ALI*: the ALI-based feature extraction network trained to minimize the standard GAN objective function (8)
- *ALI/NLL*: the proposed ALI-based feature extraction network trained to minimize the negative log-likelihood-based objective function (16)

The differential entropies are averaged in six different duration groups: less than one second (i.e., <1), 1–2 s, 2–3 s, 3–4 s, 4–5 s, and more than five seconds (i.e., >5).

As shown in the result, the differential entropies computed using the variances of the latent variables extracted from *VAE* and *ALI/NLL* gradually decrease as the duration increases. However, the differential entropy computed using the latent variable from *ALI* does not decrease dramatically compared to the other methods. This may be due to the fact that *ALI* are trained only to generate a GMM supervector similar to the one obtained via MAP adaptation (14), which is determined by the input Baum–Welch statistics. Therefore, the latent variable of *ALI* will only be able to preserve information needed for reconstructing a deterministic distribution. On the other hand, *VAE* and *ALI/NLL* is trained to generate a GMM distribution according to the maximum likelihood criterion (12), thus their latent variables may capture more information about the variability within the generated GMM distribution.

Another interesting observation from Figure 6 is that the relative decrement in entropy was much greater in *ALI/NLL* than *VAE*. While the change in entropy is rather conservative in the *VAE* case, where the relative decrement between the first duration group (i.e., less than one second) and the sixth duration group (i.e., more than five seconds) was 29.91%, the entropy in *ALI/NLL* changed dramatically with a relative decrement of 330.17%. This shows that regularizing the latent variable with a joint discriminator network is more effective than using the KL divergence-based regularization for capturing the uncertainty.

### 4.4. Speaker Verification and Identification with Different Utterance-Level Features

In this subsection, we evaluated the performance of the features extracted from various techniques. More specifically, we compared the performance of the conventional i-vector and the latent variable mean (*LM*) and log-variance (*LV*) extracted from the VAE- and ALI-based feature extractors (i.e., *VAE*, *ALI* and *ALI/NLL*). In addition, we conducted feature-level fusion between different features and evaluated their performance. For feature-level fusion, we simply concatenated the different features together to create a supervector. The i-vector features used in this experiment were:

- *i-vector(200)*: standard 200-dimensional i-vector
- *i-vector(400)*: standard 400-dimensional i-vector
- *i-vector(600)*: standard 600-dimensional i-vector

and the latent variable features were:

- *LM*: 200-dimensional latent variable mean
- *LV*: 200-dimensional latent variable log-variance

The fusion features used in this experiment were:

- *LM + LV*: concatenation of the 200-dimensional latent variable mean and the log-variance, resulting in a 400-dimensional vector
- *i-vector + LM*: concatenation of the 200-dimensional i-vector and the 200-dimensional latent variable mean, resulting in a 400-dimensional vector
- *i-vector + LV*: concatenation of the 200-dimensional i-vector and the 200-dimensional latent variable log-variance, resulting in a 400-dimensional vector
- *i-vector + LM + LV*: concatenation of the 200-dimensional i-vector and the 200-dimensional latent variable mean and log-variance, resulting in a 600-dimensional vector

**Figure 7:**
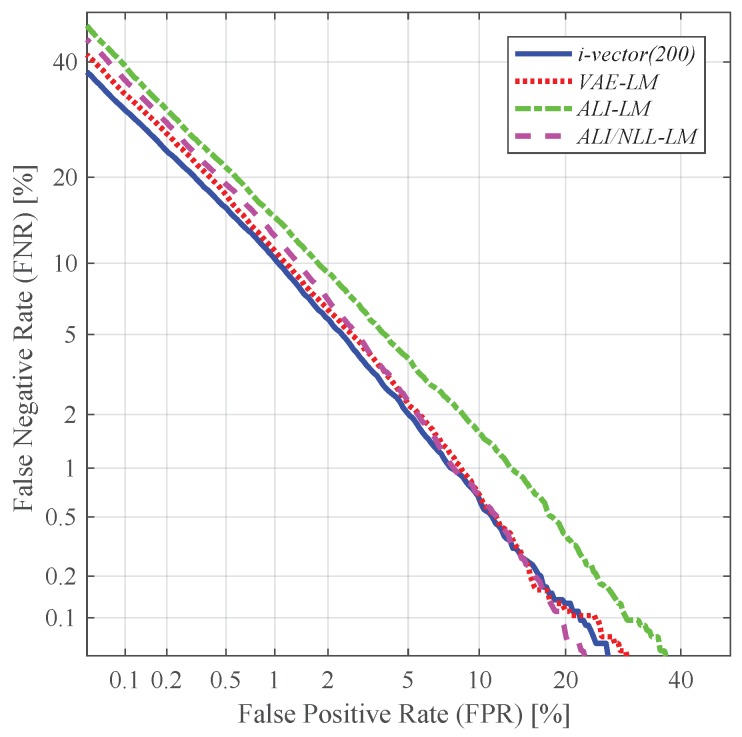
DET curves of the speaker verification experiments using latent variable mean as features.

**Figure 8:**
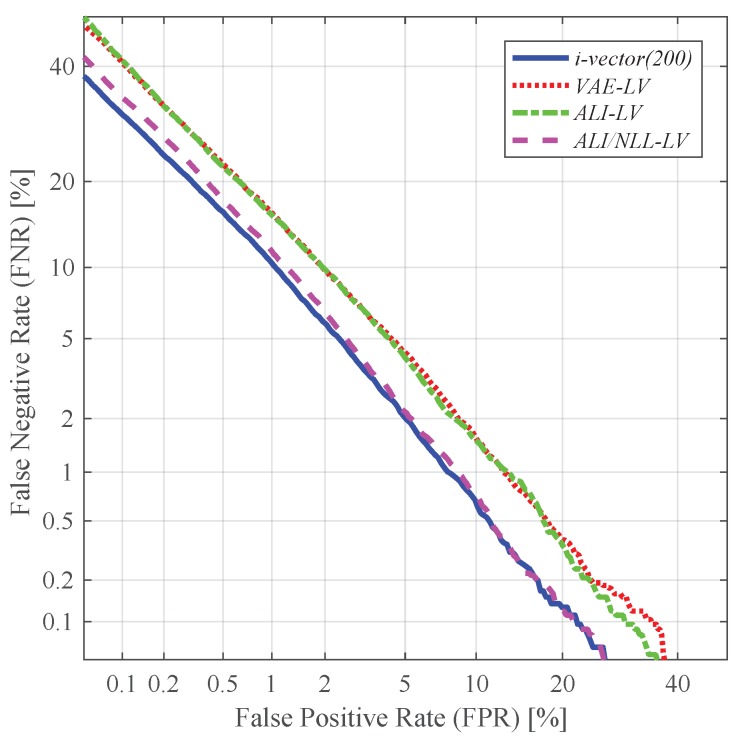
DET curves of the speaker verification experiments using latent variable log-variance as features.

**Figure 9:**
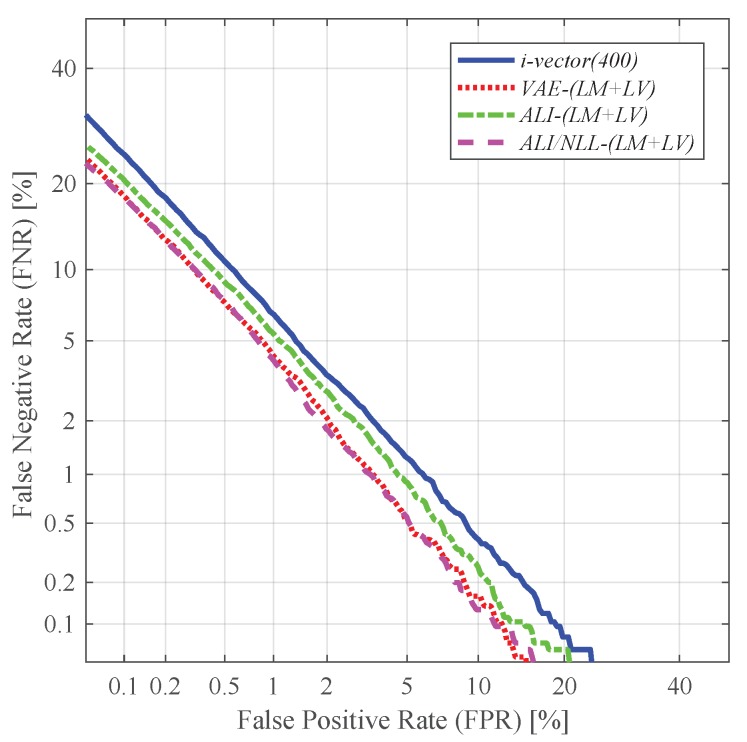
DET curves of the speaker verification experiments using the concatenation of the latent variable mean and log-variance as features.

Table 1 and Table 2 respectively give the EER and classification error results obtained by using these features. As depicted in Figure 7, the latent variable mean vector extracted from the VAE- and ALI-based feature extractors (i.e., *VAE*, *ALI*, *ALI/NLL*) shows promising performance. In particular, the performance yielded by the latent variable mean of *VAE* and *ALI/NLL* (i.e.,*VAE-LM*, *ALI/NLL-LM*) seems to be comparable to the conventional i-vector. On the other hand, as shown in Figure 8, the latent variable log-variance of *VAE* and *ALI* (i.e.,*VAE-LV*, *ALI-LV*) shows relatively poor performance compared to the latent variable mean features. However, as shown in Figure 8, the latent variable log-variance extracted from *ALI/NLL* (i.e., *ALI/NLL-LV*) outperformed the latent variable log-variance features extracted from all three networks (i.e., *VAE*, *ALI*, *ALI/NLL*) in terms of EER. This shows that the proposed network *ALI/NLL* is capable of generating latent variable variance which not only implies the uncertainty within the input speech but also encodes a sufficient amount of speaker-dependent information. Using the latent variable together (i.e., *LM + LV*) as a combined feature further improved the performance, and the best performing feature was obtained from *ALI/NLL*, which achieved a relative improvement of 28.73% in terms of EER compared to that of *i-vector (400)*. Figure 9 shows the detection error trade-off (DET) curves obtained from the experiments using *LM + LV*.

**Figure 10:**
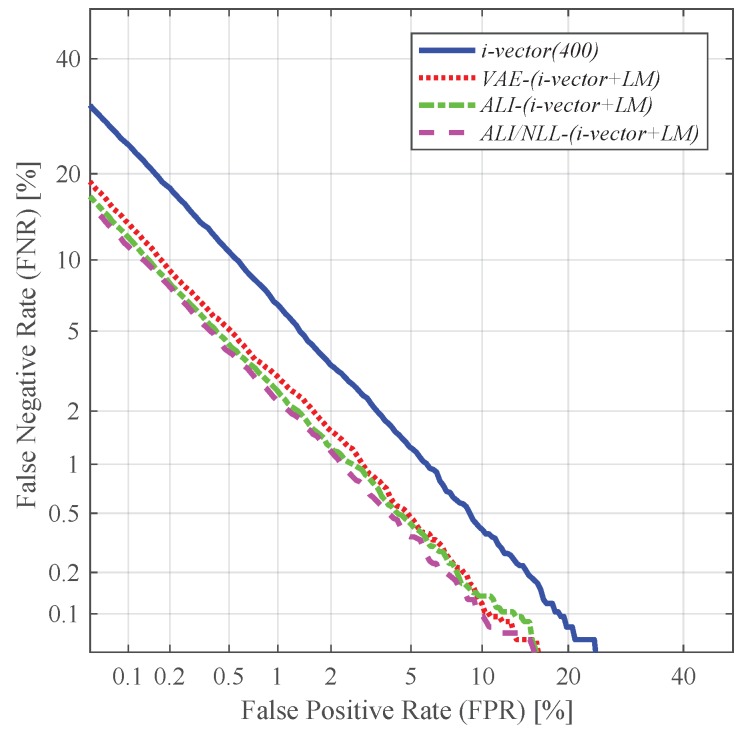
DET curves of the speaker verification experiments using the i-vectors augmented with the latent variable mean as features.

As shown in Figure 10, augmenting the standard i-vector and the latent variable mean (i.e., *i-vector + LM*) further improved the speaker verification performance. This improvement may be attributed to the nonlinear feature extraction process of the VAE- and ALI-based methods. Since the latent variable mean is trained to capture the variability within the distribution of the input utterance via a nonlinear process, it is likely to encompass information not attainable in the standard i-vector. Especially the one augmented with the latent variable mean extracted from *ALI/NLL* (i.e., *ALI/NLL-(i-vector + LM)*) showed better performance than the ones extracted from *VAE* and *ALI*, achieving a relative improvement of 42.16% in terms of EER compared to *i-vector (400)*. The reason behind this may be due to the fact that the latent variable of *ALI/NLL* can preserve the distinctive information much better by incorporating a joint discriminator instead of regularizing the latent variable distribution via KL divergence.

**Figure 11:**
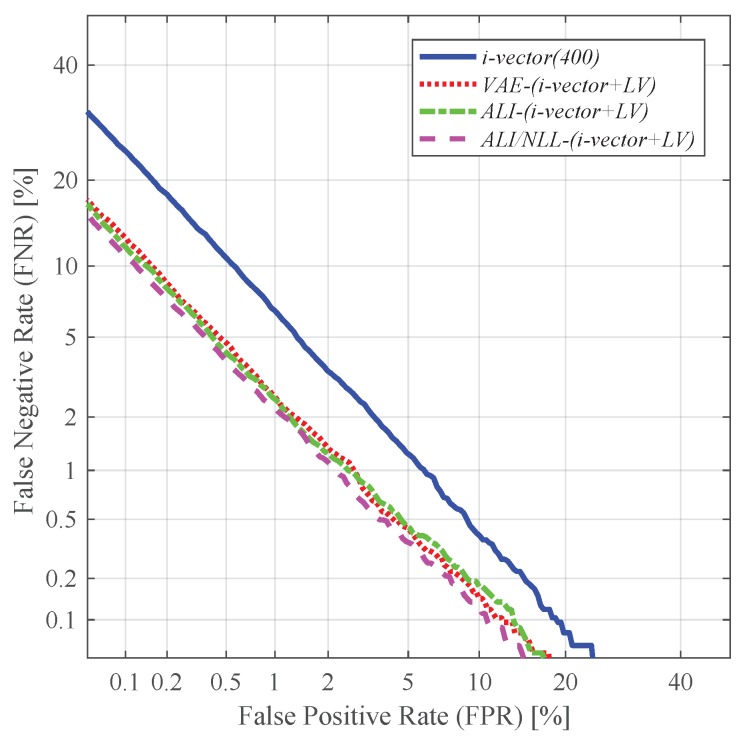
DET curves of the speaker verification experiments using the i-vectors augmented with the latent variable log-variance as features.

Likewise, using the i-vector in conjunction with the latent variable log-variance (i.e., *i-vector + LV*) also showed improvement in performance. Similar to the *i-vector + LM* experiments, the i-vector augmented with the latent variable log-variance extracted from *ALI/NLL* (i.e., *ALI/NLL-(i-vector + LV)*) outperformed the other methods (i.e., *VAE-(i-vector + LV)*, *ALI-(i-vector + LV)*), achieving a relative improvement of 34.66% compared to *i-vector (400)* in terms of EER. This may be due to the capability of the latent variable variance extracted from *ALI/NLL* in capturing the amount of uncertainty, which has been discussed in the previous subsection. Figure 11 shows the DET curves obtained from the experiments using *LV*.

**Figure 12:**
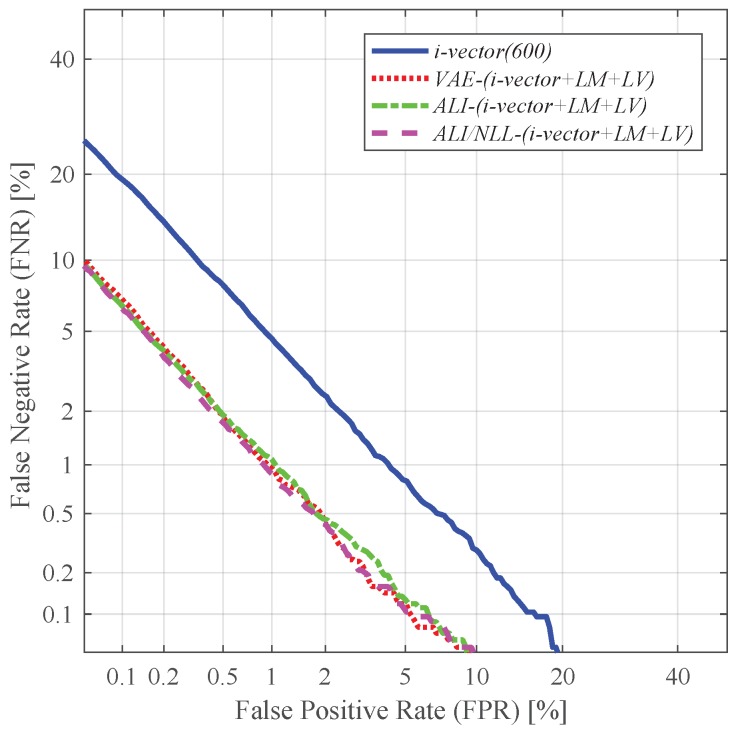
DET curves of the speaker verification experiments using the i-vectors augmented with the latent variable mean and log-variance as features.

Further improvement was observed when augmented with both the latent variable mean and log-variance (*i-vector + LM + LV*), which can be seen in Figure 12. The standard i-vector used in conjunction with the latent variable mean and log-variance extracted from *ALI/NLL* (i.e., *ALI/NLL-(i-vector + LM + LV)*) showed a relative improvement of 56.68% in terms of EER compared to the conventional i-vector with the same dimension (i.e., *i-vector(600)*).

## 5. Conclusions

In this paper, a novel utterance-level feature extractor using an adversarial learning framework for speaker recognition is proposed. Analogous to the previously proposed VAE-based feature extractor, the architecture proposed in this paper is composed of an encoder and a decoder network where the former estimates the distribution of the latent variable given the speech and the latter generates the GMM from the latent variable. However, in order to prevent the potential loss of distinctive representation for the speaker within the extracted latent variable, the newly proposed feature extractor is trained according to the ALI framework where a joint discriminator network is exploited to ensure that the latent variable and the generated GMM are close to their prior distribution and the GMM obtained through MAP adaptation, respectively.

To evaluate the performance of the features extracted from the proposed system in a short duration scenario, we conducted a set of experiments using the TIDIGITS dataset. From the results, we observed that the variance of the latent variable extracted from the proposed ALI-based feature extractor is more useful to represent the level of uncertainty caused by the short duration of the given speech than the one extracted from the VAE-based feature extractor. Moreover, using the features extracted from the proposed ALI-based method in conjunction with the standard i-vector was shown to be far more effective than the VAE-based method.

Although the proposed method showed good performance in the short duration scenario, we believe that there are still some improvements that could be made. One limitation of the proposed ALI-based method is that it relies on a pretrained GMM model (i.e., UBM), which requires an extra training phase and yields representation of the data distribution limited by the GMM structure.

Therefore, in our future study, we will expand our proposed method to jointly train the background model (i.e., UBM) along with the feature extraction network. Moreover, we will explore the effect of the uncertainty caused by environmental contamination (e.g., noise, reverberation) on the latent variable extracted from the proposed model by evaluating using more adversarial datasets (e.g., VoxCeleb, NIST SRE).

## Figures and Tables

**Table 1:** EER comparison between various feature-level fusions of the conventional i-vector, mean and log-variance of the latent variables extracted from the VAE- and ALI-based feature extractors [%].

	*LM*	*LV*	*LM+LV*	*i-Vector + LM*	*i-Vector + LV*	*i-Vector + LM + LV*
*i-vector(200)*	**3.36**					
*i-vector(400)*			2.68			
*i-vector(600)*						2.17
*VAE*	3.61	4.65	2.03	1.78	1.65	0.97
*ALI*	4.39	4.56	2.32	1.59	1.55	1.03
*ALI/NLL*	3.64	3.46	1.91	1.55	**1.51**	**0.94**

**Table 2:** Classification error comparison between various feature-level fusions of the conventional i-vector, mean and log-variance of the latent variables extracted from the VAE- and ALI-based feature extractors [%].

	*LM*	*LV*	*LM + LV*	*i-Vector + LM*	*i-Vector + LV*	*i-Vector + LM + LV*
*i-vector(200)*	12.62					
*i-vector(400)*			7.67			
*i-vector(600)*						5.07
*VAE*	**11.89**	17.78	6.94	5.36	4.99	2.75
*ALI*	16.62	17.31	8.54	5.10	4.79	2.79
*ALI/NLL*	12.56	12.38	6.76	**3.97**	4.18	**2.49**
